# Emergency Bleeding Control Interventions After Immediate Total-Body CT Scans in Trauma Patients

**DOI:** 10.1007/s00268-018-4818-0

**Published:** 2018-10-16

**Authors:** Kaij Treskes, Teun P. Saltzherr, Michael J. R. Edwards, Benn J. A. Beuker, D. Den Hartog, Joachim Hohmann, Jan S. Luitse, Ludo F. M. Beenen, Markus W. Hollmann, Marcel G. W. Dijkgraaf, J. Carel Goslings, the REACT-2 study group, J. C. Sierink, N. W. L. Schep, R. W. Peters, T. J. Tromp, M. Brink, R. van Vugt, J. S. Harbers, M. W. J. L. A. Wertenbroek, K. ten Duis, P. P. M. Rood, P. P. De Rooij, E. M. M. Van Lieshout, R. Bingisser, N. Bless, C. Zaehringer

**Affiliations:** 1 Trauma Unit, Department of Surgery, Amsterdam University Medical Center, University of Amsterdam, Meibergdreef 9, 1105 AZ Amsterdam, Netherlands; 2 Department of Surgery, Haaglanden Medical Center, Lijnbaan 32, 2512 VA Den Haag, Netherlands; 3 Trauma Unit, Department of Surgery, Radboud University Medical Center, Geert Grooteplein-Zuid 10, 6525 GA Nijmegen, Netherlands; 4 Trauma Unit, Department of Surgery, University Medical Center Groningen, Hanzeplein 1, 9700 RB Groningen, Netherlands; 5 Trauma Research Unit, Department of Surgery, Erasmus MC, University Medical Center Rotterdam, ‘s-Gravendijkwal 230, 3015 CE Rotterdam, Netherlands; 6 Department of Radiology and Nuclear Medicine, University of Basel Hospital, Petersgraben, 4031 Basel, Switzerland; 7 Department of Radiology, Amsterdam University Medical Center, University of Amsterdam, Meibergdreef 9, 1105 AZ Amsterdam, Netherlands; 8 Department of Anaesthesiology, Academic Medical Center, Meibergdreef 9, 1105 AZ Amsterdam, Netherlands; 9 Clinical Research Unit, Department of Clinical Epidemiology, Biostatistics and Bioinformatics, Amsterdam University Medical Center, University of Amsterdam, Meibergdreef 9, 1105 AZ Amsterdam, Netherlands; 10 Department of Surgery, Onze Lieve Vrouwe Gasthuis, Jan Tooropstraat 164, 1061 AE Amsterdam, Netherlands

## Abstract

**Background:**

Immediate total-body CT (iTBCT) is often used for screening of potential severely injured patients. Patients requiring emergency bleeding control interventions benefit from fast and optimal trauma screening. The aim of this study was to assess whether an initial trauma assessment with iTBCT is associated with lower mortality in patients requiring emergency bleeding control interventions.

**Methods:**

In the REACT-2 trial, patients who sustained major trauma were randomized for iTBCT or for conventional imaging and selective CT scanning (standard workup; STWU) in five trauma centers. Patients who underwent emergency bleeding control interventions following their initial trauma assessment with iTBCT were compared for mortality and clinically relevant time intervals to patients that underwent the initial trauma assessment with the STWU.

**Results:**

In the REACT-2 trial, 1083 patients were enrolled of which 172 (15.9%) underwent emergency bleeding control interventions following their initial trauma assessment. Within these 172 patients, 85 (49.4%) underwent iTBCT as primary diagnostic modality during the initial trauma assessment. In trauma patients requiring emergency bleeding control interventions, in-hospital mortality was 12.9% (95% CI 7.2–21.9%) in the iTBCT group compared to 24.1% (95% CI 16.3–34.2%) in the STWU group (*p* = 0.059). Time to bleeding control intervention was not reduced; 82 min (IQR 5–121) versus 98 min (IQR 62–147), *p* = 0.108.

**Conclusions:**

Reduction in mortality in trauma patients requiring emergency bleeding control interventions by iTBCT could not be demonstrated in this study. However, a potentially clinically relevant absolute risk reduction of 11.2% (95% CI − 0.3 to 22.7%) in comparison with STWU was observed.

**Trial registration:**

ClinicalTrials.gov: NCT01523626.

## Background

Improvements in speed and accuracy of computed tomography (CT) make immediate total-body CT (iTBCT) feasible as a diagnostic tool in the primary care for severe trauma patients. iTBCT scanning in trauma patients is safe, shortens the time to end of diagnostic imaging and does not increase direct medical costs [1]. However, it does not improve survival in the total group of severe trauma patients [1]. Which patients exactly could benefit from this fast and detailed diagnostic approach remains unclear.

Patients requiring emergency bleeding control interventions benefit from fast and optimal trauma screening, obtaining as much information on the bleeding site(s) as is safely possible. iTBCT during the initial trauma assessment might improve survival in this specific patient group. Time to surgery is reported to be shorter for patients requiring emergency surgery after total-body CT scanning [2]. Potential survival benefits associated with total-body CT scanning in severely injured patients requiring bleeding control measurements have been described previously [3].

The aim of this study was to assess whether an initial trauma screening with iTBCT is associated with lower in-hospital mortality and shorter clinically relevant time intervals in patients requiring emergency bleeding control interventions compared to trauma screening with conventional imaging and selective CT scanning of specific body regions.

## Methods

### Study design and patient selection

In the REACT-2 trial, non-pregnant patients, 18 years and over, who sustained a major trauma, were included on compromised vital parameters, clinical suspicion of specific severe injuries or high-risk trauma mechanism in five trauma centers in the Netherlands and Switzerland between April 21, 2011 and January 1, 2014. Patients were considered eligible when meeting one or more of the inclusion criteria and none of the exclusion criteria shown in Table 4 of the Appendix.

Patients were randomized for iTBCT or conventional imaging with selective CT of specific body regions. Decision of eligibility by the trauma leader as well as documentation of the indication by a trauma team member was performed before the start of radiologic imaging. Potential life-saving interventions were performed prior to radiologic imaging when indicated, e.g., endotracheal intubation or chest tube placement. iTBCT was performed without preceding conventional imaging and consisted of an unenhanced CT of the head and neck and a contrast enhanced CT of thorax, abdomen and pelvis. The design of the REACT-2 study has been previously described (ClinicalTrials.gov: NCT01523626) and published [1, 4]. The REACT-2 study was approved by the medical research ethics committees at all participating centers (AMC MEC 10/145).

For this study, patients who underwent emergency bleeding control interventions following their initial trauma assessment were selected for further analysis. Emergency bleeding control interventions were defined as thoracotomy, laparotomy, external fixation of the pelvis or extremities and angiographic embolization. Multitrauma patients were defined by an Injury Severity Score (ISS) ≥ 16 for an exploratory subgroup analysis. In addition to the intention-to-treat analysis, a per-protocol analysis was performed in which crossovers (i.e., patients who received the opposite intervention to which they had been allocated) were excluded.

Time intervals were prospectively recorded and started as the patient arrived in the trauma resuscitation room. Time to end of imaging was defined as the time from arrival in the trauma room to the end of imaging of the initial trauma assessment. Time to diagnosis was defined as the time from arrival to the time all life-threatening injuries were diagnosed according to the trauma team leader. Time at the ED (emergency department) was defined by the time of arrival to the time of departure from the trauma room. Time to intervention was defined by the time of arrival to the time an emergency bleeding control intervention was initiated. Hypotension was defined as systolic blood pressure below 90 mmHg.

### Statistical analysis

Continuous data with a normal distribution are presented as means and standard deviations. The non-normally distributed data are presented as medians with interquartile range. Independent sample t tests and Mann–Whitney *U* tests were used to compare the parametric and nonparametric continuous data, respectively. The Chi-squared test or Fisher’s exact test was used to compare the categorical variables. The 95% confidence intervals for proportions were calculated with the modified Wald method. A *p* value of less than 0.05 was considered statistically significant. All statistical analyses were performed with SPSS version 24 (SPSS inc., Chicago, Illinois).

## Results

In the REACT-2 trial, 1083 patients were enrolled of which 172 (15.9%) underwent emergency bleeding control interventions directly following their initial trauma assessment. Within these 172 patients, 85 (49.4%) underwent iTBCT as primary diagnostic modality. Median ISS was 27 (IQR 20–41) in the iTBCT group compared to 29 (IQR 18–41) in the standard workup (STWU) group (*p* = 0.994). Hypotension at admission was present in 21.7% of the iTBCT group compared to 20.0% in the STWU group (*p* = 0.788). Baseline demographic and clinical characteristics are presented in Table 1.

**Table 1:** Demographic and clinical characteristics*

Characteristic	Total-body CT (*n* = 85)	Standard workup (*n* = 87)
Age (years)^a^	41 (26–56)	46 (28–60)
Male sex, *n* (%)	69 (81.2)	66 (75.9)
Blunt trauma, *n* (%)	82 (96.5)	85 (97.7)
Comorbidity, *n* (%)		
ASA I or II	78 (96.3)	79 (97.5)
ASA III, IV or V	3 (3.7)	2 (2.5)
In-hospital vital parameters		
Respiratory rate (per minute)^a^	16 (14–20)	16 (14–20)
Pulse (bpm)^b^	99 (20)	95 (26)
Systolic blood pressure (mmHg)^b^	117 (28)	115 (28)
GCS (points)^a^	11 (3–15)	11 (3–15)
Revised Trauma Score^a^	7.11 (4.09–7.84)	6.90 (4.09–7.84)

*ASA* American Society of Anaesthesiologists

*p > 0.05 for all between-group comparisons

^a^Median (interquartile range)

^b^Mean (SD)

In 85 patients in the iTBCT group, 108 emergency bleeding control interventions were performed. In the STWU group, 109 emergency bleeding control interventions were performed in 87 patients. In the iTBCT group, more patients underwent external fixations of the extremities than in the STWU group (56.5 vs. 40.2%, *p* = 0.033). Injury severity parameters and surgical characteristics are presented in Table 2.

**Table 2:** Injury severity and surgical characteristics*

Characteristic	Total-body CT (*n* = 85)	Standard workup (*n* = 87)
Abbreviated Injury Scale ≥ 3, *n* (%)		
Head	37 (43.5)	32 (36.8)
Chest	52 (61.2)	51 (49.5)
Abdomen	27 (31.8)	38 (43.7)
Extremities	62 (72.9)	57 (65.5)
Emergency interventions, *n* (%)	108	109
Thoracotomy	7 (8.2)	6 (6.9)
Laparotomy	20 (23.5)	32 (36.8)
External fixation of the pelvis	19 (22.4)	19 (21.8)
External fixation of extremities	48 (56.5)	35 (40.2)
Angiographic embolization	14 (16.5)	17 (19.5)
Injury Severity Score (points)	27 (20–41)	29 (18–41)
Multitrauma patients, *n* (%)^a^	75 (88.2)	72 (82.8)
TBI patients, *n* (%)^a^	29 (34.1)	24 (27.6)
TRISS, survival probability	0.84 (0.30–0.97)	0.89 (0.48–0.98)

Data are number (%) or median (interquartile range)

*TRISS* Trauma and Injury Severity Score

*p > 0.05 for all between-group comparisons except for external fixation of extremities (*p* = 0.033)

^a^Multitrauma patients are defined as ISS ≥ 16. Traumatic brain injury (TBI) patients are defined as GCS < 9 at presentation and AIS Head ≥ 3

**Fig. 1:**
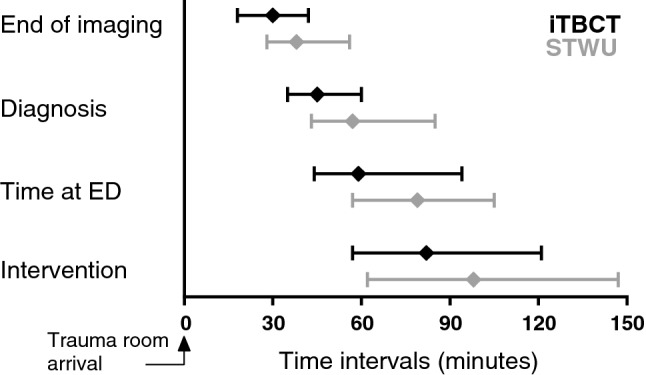
Clinically relevant time intervals. Medians and interquartile ranges of clinically relevant time intervals (minutes) are displayed per randomisation group. *p* < 0.05 for time to end of imaging, time to diagnosis and time at ED. *iTBCT* immediate total-body CT, *STWU* standard workup, *ED* emergency department

In-hospital mortality was 12.9% (95% CI 7.2–21.9%) in the iTBCT group compared to 24.1% (95% CI 16.3–34.2%) in the STWU group (absolute risk reduction: 11.2%, 95% CI − 0.3 to 22.7%; *p* = 0.059). Time to diagnosis was reduced for patients who underwent iTBCT: 45 min (IQR 35–60) versus 57 min (IQR 43–85), *p* = 0.009. Time to bleeding control intervention was not reduced: iTBCT 82 min (IQR 57–121) versus STWU 98 min (IQR 62–147), *p* = 0.108. Outcomes for patients requiring emergency bleeding control interventions are presented in Table 3 and time intervals are displayed in Fig. 1.

**Table 3:** Outcome for patients requiring emergency bleeding control interventions

Characteristic	Total-body CT (*n* = 85)	Standard workup (*n* = 87)	*p* value
Mortality; *n*, % (95% CI)			
In-hospital mortality	*n* = 11 12.9% (7.2–21.9)	*n* = 21 24.1% (16.3–34.2)	0.059*
24-h mortality	*n* = 4 4.7% (1.5–11.9)	*n* = 6 6.9% (2.9–14.5)	0.747^†^
Time intervals, minutes (IQR)			
Time to end of imaging	30 (18–42)	38 (28–56)	0.006
Time to diagnosis	45 (35–60)	57 (43–85)	0.009^‡^
Time at ED	59 (44–94)	79 (57–105)	0.041^‡^
Time to intervention	82 (57–121)	98 (62–147)	0.108^‡^
Complications; *n*, % (95% CI)	*n* = 39 45.9% (35.7–56.4)	*n* = 42 48.3% (38.1–58.6)	0.753*
Length of stay, days (IQR)			
Total hospital stay	23 (12–37)	20 (10–33)	0.606^‡^
ICU stay	5 (2–12)	6 (2–12)	0.909^‡^
Ventilation days	3 (1–9)	3 (1–8)	0.928^‡^

Data are number, % (95% confidence interval by modified Wald) or median (interquartile range)

*OR* odds ratio, *CI* confidence interval, *ED* emergency department

*Chi-squared test, ^†^Fisher’s exact test, ^‡^Mann–Whitney *U* test

In an exploratory analysis in the group of multitrauma patients, in-hospital mortality was reduced after iTBCT compared to the STWU group: 13.3% (95% CI 7.2–23.0%) versus 27.8% (95% CI 18.7–39.1%), with an absolute risk reduction of 14.4% (95% CI 1.6–27.3%, *p* = 0.030). Time to diagnosis was reduced for patients who underwent iTBCT: 47 min (IQR 35–61) versus 57 min (IQR 42–83), *p* = 0.033. Time to bleeding control intervention was not reduced: iTBCT 78 min (IQR 56–120) versus STWU 92 min (IQR 62–125), *p* = 0.306. Outcomes for multitrauma patients (ISS ≥ 16) requiring emergency bleeding control interventions are presented in Table 5 of the Appendix.

In the per-protocol analysis, two crossovers were excluded. No relevant differences in outcome were found for all endpoints in comparison with the original intention-to-treat analysis as shown in Table 6 of the Appendix. With multivariate analyses on in-hospital mortality corrected for center and type of intervention and analyses on time to intervention stratified for center and type of intervention, no relevant differences were found in comparison with the original analyses.

## Discussion

This study could not demonstrate a beneficial effect on survival of iTBCT for trauma patients requiring emergency bleeding control interventions. However, a potentially clinically relevant absolute risk reduction of 11.2% (95% CI − 0.3 to 22.7%) in comparison with STWU was observed. The original study had been powered to detect an absolute risk reduction of 5% (from 12 to 7%) in severe trauma patients, irrespective of their need for emergency bleeding control interventions, but was underpowered for the analysis in the subgroup requiring such intervention.

The potential reduction in mortality by iTBCT after major trauma could be the effect of a faster trauma workup. In addition, the complete information by iTBCT before treatment could sharpen the indication of the intervention and help the team to prepare and prioritize in the case of multiple targets for interventions. This hypothesis is supported by the study from Wada et al. [3] who reported reduced mortality for patients receiving TBCT before emergency bleeding control measurements in a retrospective study in two trauma centers. In contrast to Wada et al. [3], and Wurmb et al. [2] report unchanged mortality by TBCT for patients requiring any surgery immediately after resuscitation in multiple trauma patients in a retrospective single-center study. However, they concluded that an improvement in outcome might be assumed since the patients receiving TBCT were more severely injured [2]. This difference in injury severity could be explained by the use of a triage scheme for the TBCT group, selecting more severely injured patients for TBCT.

Huber-Wagner et al. [5] report reduced mortality for trauma patients in moderate and severe shock that underwent TBCT in a large retrospective multicenter study. Ordonez et al. report no mortality reduction in hemodynamically unstable trauma patients after CT, however, did report a survival benefit for hemodynamically unstable patients with an ISS ≥ 25 in a single-center retrospective study. Furthermore, they report changes in indication and planning for surgery in a substantial part of the patients [6]. This further supports the use of TBCT for severely injured bleeding patients requiring fast treatment.

The relationship between iTBCT and mortality could be further supported if we could demonstrate not only a reduction in time to diagnosis but also a reduction in time to intervention. Several studies did find a benefit for time to intervention after TBCT in retrospective studies [2, 3, 7]. In the present study, there was a wide range of time to intervention intervals which could be the effect of potential confounders as center of treatment and/or different intervention types. Analyses on time to intervention stratified for center and type of intervention did not show differences compared to the original analyses.

The decision to perform an iTBCT is based on information obtained during the pre-hospital phase and during the in-hospital primary survey. Criteria for TBCT in trauma patients are diverse [8], and often the imaging itself is needed for the identification of a severely injured patient with the necessity for emergency bleeding control interventions. Selecting the appropriate patients for iTBCT and minimizing radiation exposure for the less severely injured patients remain a challenge.

A limitation of our study is that this subgroup analysis was unplanned at the design stage, resulting in a lack of statistical power for the detection of the observed clinically relevant contrast between the mortality rates. During the enrollment of our trial, associations between TBCT and emergency bleeding control interventions were reported and made this subgroup of specific interest and therefore legitimize the additional analysis on these patients. Strength of this multicenter study is the assessment of a prospectively enrolled and randomized population. Further research should be performed to confirm the suggested reduction in mortality by iTBCT in trauma patients requiring bleeding control interventions. Furthermore, future research should focus on how to select patients who could benefit from iTBCT after trauma.

## Conclusion

This study could not demonstrate a beneficial effect on survival by the fast and detailed diagnostic workup by immediate total-body CT for trauma patients requiring emergency bleeding control interventions. There is probably a lack of statistical power for the detection of the potentially clinically relevant risk reduction in mortality by iTBCT. Further research should be performed to confirm the suggested reduction in mortality by iTBCT in trauma patients requiring bleeding control interventions.

## Appendix

See Tables 4, 5 and 6.

**Table 4:** Criteria for immediate total-body CT in trauma patients used in REACT-2 trial

Trauma patients with one of the following parameters at hospital arrival:
• respiratory rate ≥ 30/min or ≤ 10/min
• pulse ≥ 120/min
• systolic blood pressure ≤ 100 mmHg
• estimated exterior blood loss ≥ 500 ml
• Glasgow coma score ≤ 13
*OR*
Patients with a clinical suspicion of one of the following diagnoses:
• fractures from at least two long bones
• flail chest, open chest or multiple rib fractures
• severe abdominal injury
• pelvic fracture
• unstable vertebral fractures/spinal cord compression
*OR*
Patients with one of the following injury mechanisms:
• fall from a height (> 3 m/> 10 feet)
• ejection from a vehicle
• death of occupant in same vehicle
• severely injured patient in same vehicle
• wedged or trapped chest/abdomen
*Contra-indications*
Trauma patients with one of the following characteristics were excluded:
• known age < 18 years
• known pregnancy
• referred from another hospital
• clearly low-energy trauma with blunt injury mechanism
• any patient with a stab wound in one body region
• any patient who is judged to be too unstable to undergo a CT scan and requires (cardiopulmonary) resuscitation or immediate operation because death is imminent

**Table 5:** Outcome for multitrauma patients (ISS ≥ 16) requiring emergency bleeding control interventions

Characteristic	Total-body CT (*n *= 75)	Standard workup (*n *= 72)	*p* value
Mortality; *n*, % (95% CI)			
In-hospital mortality	*n* = 10 13.3% (7.2–23.0)	*n* = 20 27.8% (18.7–39.1)	0.030*
24-h mortality	*n* = 3 4.0% (0.9–11.6)	*n* = 6 8.3% (3.6–17.3)	0.320^†^
Time intervals, minutes (IQR)			
Time to end of imaging	30 (17–42)	38 (27–56)	0.019^‡^
Time to diagnosis	47 (35–61)	57 (42–83)	0.033^‡^
Time at ED	65 (45–99)	79 (57–107)	0.139^‡^
Time to intervention	78 (56–120)	92 (62–125)	0.306^‡^
Complications; *n*, % (95% CI)	*n* = 38 50.7% (39.6–61.7)	*n* = 38 52.8% (41.4–63.9)	0.798*
Length of stay, days (IQR)			
Total hospital stay	23 (12–40)	21 (10–35)	0.640^‡^
ICU stay	6 (2–14)	6 (2–14)	0.910^‡^
Ventilation days	4 (1–9)	4 (1–8)	0.968^‡^

Data are number, % (95% confidence interval by modified Wald) or median (interquartile range)

*OR* odds ratio, *CI* confidence interval, *ISS* Injury Severity Score, *ED* emergency department

*Chi-squared test; ^†^Fisher’s exact test; ^‡^Mann–Whitney *U* test

**Table 6:** Outcome by per-protocol analysis for patients requiring emergency bleeding control interventions

Characteristic	Total-body CT (*n *= 84)	Standard workup (*n *= 86)	*p* value
Mortality; *n*, % (95% CI)			
In-hospital mortality	*n* = 11 13.1% (7.3–22.1)	*n* = 21 24.4% (16.5–34.5)	0.059*
24-h mortality	*n* = 4 4.8% (1.5–12.0)	*n* = 6 7.0% (3.0–14.7)	0.747^†^
Time intervals, minutes (IQR)			
Time to end of imaging	30 (18–42)	38 (28–57)	0.008^‡^
Time to diagnosis	45 (35–60)	57 (43–85)	0.008^‡^
Time at ED	59 (44–94)	82 (57–105)	0.033^‡^
Time to intervention	82 (62–121)	96 (62–135)	0.230^‡^
Complications; *n*, % (95% CI)	*n* = 39 46.4% (36.2–57.0)	*n* = 42 48.8% (38.6–59.2)	0.753*
Length of stay, days (IQR)			
Total hospital stay	23 (12–37)	21 (10–33)	0.612^‡^
ICU stay	6 (2–12)	6 (2–13)	0.861^‡^
Ventilation days	3 (1–9)	4 (1–8)	0.939^‡^

Data are number, % (95% confidence interval by modified Wald) or median (interquartile range)

*CI* confidence interval, *ED* emergency department

*Chi-squared test; ^†^Fisher’s exact test; ^‡^Mann–Whitney *U* test
